# HIV/AIDS knowledge and attitudes assessment among women of child-bearing age in South Sudan: Findings from a Household Survey

**DOI:** 10.1371/journal.pone.0243969

**Published:** 2020-12-14

**Authors:** William Mude, Victor M. Oguoma, Hailay Abrha Gesesew, Edward K. Ameyaw, Carolyne Njue, Tafadzwa Nyanhanda, Adeniyi O. Adeleye, Tinashe Dune, Billingsley Kaambwa, Lillian Mwanri

**Affiliations:** 1 School of Health, Medical and Applied Sciences, Central Queensland University, Sydney, Australia; 2 Health Research Institute, University of Canberra, Canberra, Australia; 3 College of Medicine and Public Health, Flinders University, Adelaide, Australia; 4 School of Health Sciences, Mekelle University, Mekelle, Ethiopia; 5 The Australian Centre for Public and Population Health Research (ACPPHR), School of Public Health, University of Technology Sydney, Sydney, Australia; 6 School of Health, Medical and Applied Sciences, Central Queensland University, Melbourne, Australia; 7 School of Nursing, Midwifery & Social Sciences, Central Queensland University, MacKay, Australia; 8 School of Health Sciences, Western Sydney University, Sydney, Australia; Columbia University - MSPH, ZIMBABWE

## Abstract

This study assessed the determinants that shape HIV knowledge and attitudes among South Sudanese women by analysing a Multiple Indicator Cluster Survey collected from 9,061 women in 9,369 households. Generalised linear mixed model regression was performed. Fifty percent of respondents were aware of HIV/AIDS, with 21% and 22% exhibiting good knowledge and positive attitudes towards people with HIV/AIDS, respectively. When controlled for individual and community-level variables, younger women (AOR = 1.28, 95% CI: 1.01–162), women with primary (AOR = 2.19; 95% CI: 1.86–2.58) and secondary (AOR = 4.48; 95% CI: 3.38–5.93) education, and those living in urban areas (AOR = 1.40; 95% CI: 1.12–1.76) had significantly good knowledge. Women in the richer (AOR = 1.60; 95% CI: 1.08–2.36) and the richest (AOR = 2.02; 95% CI: 1.35–3.02) wealth quintiles had significant positive attitudes towards people with HIV/AIDS. Well-designed social and behavioural campaigns targeting uneducated women and those living in rural and remote settings will enhance knowledge of perceived risk, awareness, and ability to carry out preventive behaviours.

## Introduction

Women in South Sudan are disproportionately affected by human immunodeficiency virus/acquired immune deficiency syndrome (HIV/AIDS) [1]. In 2018, approximately 55.6% of adults (aged 15 years and older) living with HIV/AIDS in South Sudan were women [2]. Cases of new infections in young women were about one and half times more than in young men [2]. Women in South Sudan have fewer opportunities for education, employment, social and political participation [3]. Lack of access to health information, disparities in sexual relationships and marriages, financial reliance on male partners, and traditional patriarchy are factors that facilitate gender inequalities and women’s vulnerability to HIV/AIDS [3, 4].

In 2014, the Joint United Nations Programme on AIDS (UNAIDS) and partners set the 90-90-90 targets to ensure that by 2020, 90% of people living with HIV would know their HIV status, 90% of people diagnosed with HIV would access treatments, and 90% of people on antiretroviral treatment would suppress viral loads [5]. Although there have been efforts in sub-Saharan Africa (SSA) towards achieving the UNAIDS 90-90-90 targets by 2020, little progress has been made to address low HIV/AIDS knowledge among women [6]. A recent finding shows that mother to child transmission knowledge is still low among women in SSA over the last twenty years [7]. The authors reported that while women understood the prevention of HIV/AIDS, they lacked understanding about mother to child transmission. In particular, women with low education, low economic status, and living in rural areas were more likely to lack knowledge about HIV/AIDS [8]. These issues have impeded the progress towards the UNAIDS 90-90-90 targets. In South Sudan, the lack of progress towards the achievement of these targets has even lagged further behind because of civil conflicts [1, 2].

Lack of HIV/AIDS knowledge can have serious implications for public health interventions to control infection, promote testing, and engage in treatment and care [9, 10]. Despite the efforts to improve HIV/AIDS understanding, there is still stigma and discrimination against people with HIV/AIDS in South Sudan [10]. Stigma and discrimination against people with HIV/AIDS inhibit testing, which hinders the achievement of the first UNAIDS target of testing 90% of people with HIV/AIDS by 2020 [11].

Understanding attitudes toward people with HIV/AIDS is vital to support public health measures to control infection. They promote healthy behaviours such as acceptance of condom use, reduced sexual partners, testing, and engagement in treatment and support seeking [9]. A better understanding of HIV/AIDS in the community can help to plan targeted interventions, adequately use resources, and redress inequity in society by reaching the most vulnerable groups [7]. Knowledge of HIV/AIDS among women is critical to prevent mother to child transmission. Additionally, combating HIV/AIDS is one of the Sustainable Development Goals (SDG 3) and calls for empowering individuals with HIV/AIDS knowledge to protect themselves [12].

To this end, gaps still exist in the literature on knowledge and attitudes toward people with HIV in different communities around the world. For example, HIV/AIDS knowledge is still low among women in South Sudan, and only 9.8% of women aged 15–24 can correctly identify its sexual means of transmission [2]. There is a need to explore the knowledge and attitudes towards people with HIV/AIDS in South Sudan to support public health measures and redress inequitable health information. We aim to address this gap in the literature by examining HIV/AIDS knowledge and attitudes towards people with HIV/AIDS among women in South Sudan. This information is vitally important to guide public health measures and empower women in South Sudan with HIV/AIDS knowledge. This paper will provide an important reference for future projects relating to HIV/AIDS knowledge and attitudes towards people with HIV/AIDS among women in South Sudan.

## Methods

### Study design and population

**Map 1:**
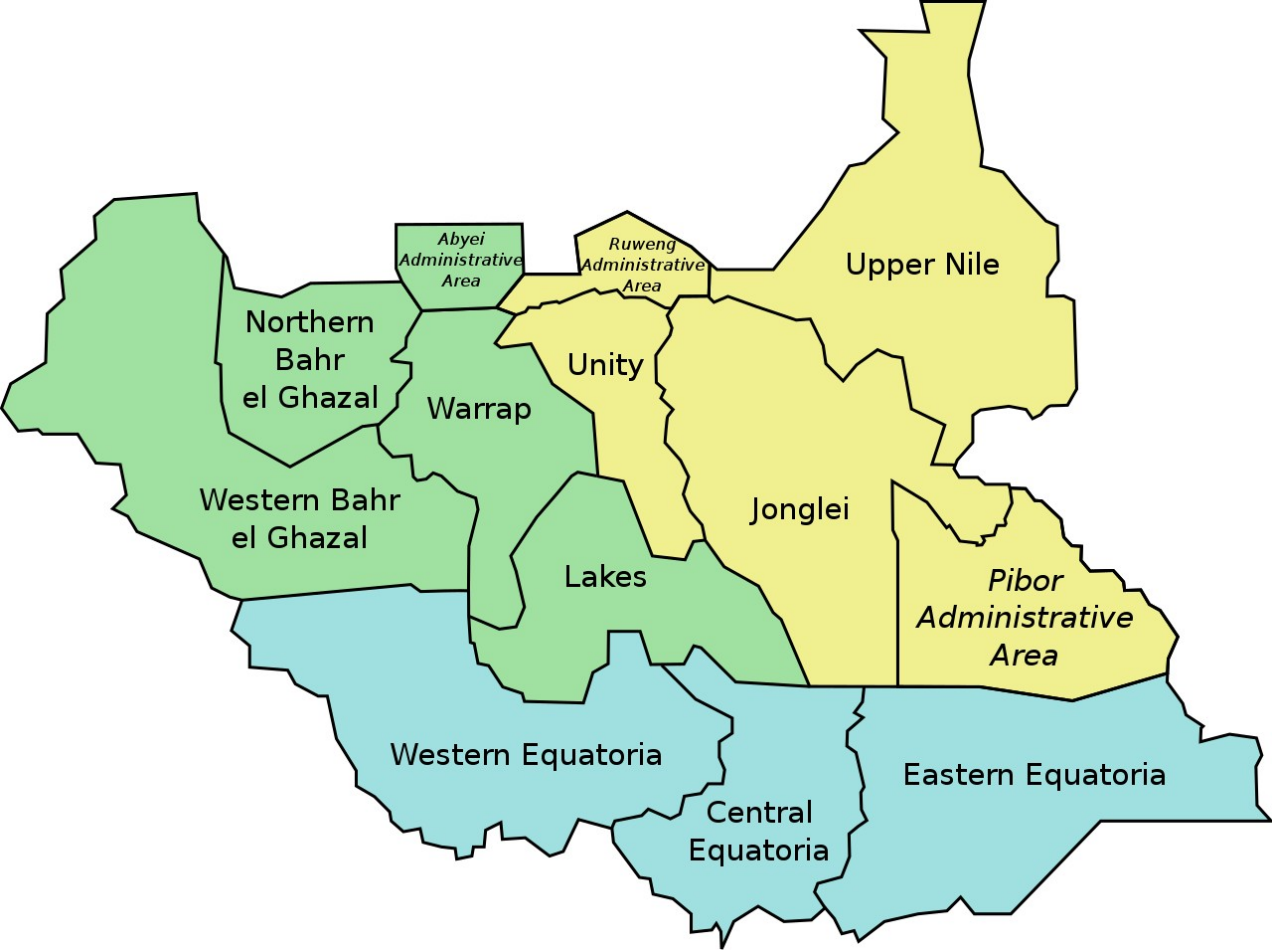
The ten states of South Sudan from where the sample was drawn. Source: Wikipedia.

We analysed data for the Multiple Indicators Cluster Surveys (MICS) for South Sudan, known as South Sudan Household Health Survey 2 (SSHHS 2). The SSHHS 2 was conducted between March and June 2010 across all the ten states of South Sudan to assess health outcome indicators among people aged between 15 and 49 years. The ten states of South Sudan where the sample was drawn from are shown in Map 1. SSHHS 2 is the only and the last large data collected at a national level. Not many HIV/AIDS awareness and programs have been implemented in the last ten years in South Sudan because of the civil wars. The design for the SSHHS 2 was based on the methodology of the MICS 4 [13], which used a two-stage cross-sectional study sampling. Twenty urban and rural areas served as the sampling strata, each state having two strata.

The survey selected 40 clusters, “enumeration areas”, from each state and 25 households per cluster. However, for an unforeseen reason, 39 clusters were drawn from each ‘Jonglei’ and ‘Unity’ states. This meant 975 households were selected from each of the two states. The total final samples included 9,950 households from 398 clusters. Further information about the sample size for the survey, sampling process, data collection techniques, and data quality measurements has been reported elsewhere [14].

The survey identified 9,950 random households of which 9,760 were occupied. Of the occupied households, 9,369 completed interviewer-administered face-to-face interviews, a 96% response rate. The interviewed respondents within each household were 9,069 women aged 15–49 years old (14). For this paper, we analysed health indicators relating to HIV/AIDS knowledge and attitudes towards people with HIV/AIDS among women of childbearing age (15–49 years old) in South Sudan.

### Study variables and measurements

#### Individual or household level covariates

Age, marital status, wealth index quintiles, and education of respondents were individual or household covariates as defined by the literature [15].

#### Community-level covariates

Place of residence (urban and rural) and the ten states comprising South Sudan were community-level variables.

#### Outcome variables

HIV knowledge and attitudes towards people with HIV/AIDS were the outcome variables. A nine scale questionnaire on HIV knowledge and a three scale questionnaire on attitudes towards people with HIV/AIDS have previously been used in HIV research literature [16].

The questionnaires asked nine statements to assess HIV knowledge in the context of South Sudan. Only respondents who indicated that they had heard of HIV/AIDS before this survey (that is, answered yes to the first statement) were asked to provide a ‘yes’ or ‘no’ response to the following remaining eight statements: (i) HIV can be avoided by having one uninfected partner, (ii) HIV can be avoided by using a condom correctly every time, (iii) People can get HIV through supernatural means, (iv) People can get HIV from mosquito bites, (v) People can get HIV by sharing food with a person who has HIV, (vi) HIV can transfer from mother to child during delivery, (vii) HIV can transfer from mother to child during pregnancy, and (viii) HIV can transfer from mother to child through breastfeeding.

A correct response was scored as one (1) and an incorrect one as zero (0) [17]. Respondents with at least six correct scores (i.e. ≥ 70% correct responses) were considered to have good knowledge about HIV while those with scores of less than 70% were considered not to have good knowledge [18, 19]. Some of the reasons for scores less than 70% were gross misconceptions about HIV transmission, guessing, and just generally inadequate knowledge about HIV [18].

Attitudes towards people with HIV/AIDS were assessed by asking respondents to state ‘yes’ or ‘no’ to the following five statements: (i) Am willing to buy fresh vegetables from a shopkeeper with HIV, (ii) A female teacher with HIV should be allowed to teach school children, (iii) Would like HIV status of a family member to remain secret, (iv) The healthy-looking person may have the AIDS virus, and (v) I am willing to care for a person with AIDS in a household.

### Statistical analysis

Descriptive statistics were calculated using frequencies and percentages for categorical variables. The ‘complex samples procedure’ in IBM SPSS 26 for Windows (IBM Corp, Armonk, NY), which accounts for survey-specific procedures for clustering, stratification, and weighting was used in the calculation of basic statistics.

A generalised linear mixed model with a logit link function accounting for sampling weight was used to assess the relationship between knowledge and attitudes towards HIV/AIDS on one hand and individual and community level covariates on the other. The model contains fixed effects for the covariates (level 1) and a random intercept-fixed slope for the cluster (level 2 –variables were the place of residence and state).

We fitted a univariate model for each level 1 covariates and outcomes to assess the relationship between each covariate and outcome. Before fitting multivariable models, a null model (an unconditional model without any covariate) was fitted first to decompose the total variance of HIV knowledge and attitudes between the cluster and level 1 covariates. Model 1 is a multivariable regression of the individual or household level factors, while model 2 further controlled for both the individual and community level factors. For the univariate analyses, estimates of the association between outcomes and individual or community level covariates are reported as odds ratio (OR) and the 95% CI. The multivariable models (Models 1 and 2) are reported as adjusted odds ratio (AOR) and 95% CI.

The analyses also explored the influence of the random effects on the likelihood of HIV knowledge and attitudes among women. The probable cluster random effects are expressed and reported as the area variance (AV), intra-cluster correlation (ICC) [20], median odds ratio (MOR) [21] and the proportion of change in variance (PCV) [22]. We assessed the goodness of fit for the multivariable models using the deviance residual and Akaike Information Criteria (AIC). The model with the smallest deviance or AIC was taken as the best fitting model. In all analyses, statistical significance was set at p<0.05. The findings were discussed and guided by the World Health Organisation Social Determinants of Health framework [23].

### Ethical considerations

For the primary research, the United Nations International Children Education Fund (UNICEF) received ethics approval from the South Sudanese Ministry of Health and the National Bureau of Statistics. Study participants provided informed consent, and the anonymity of information was protected. For the current study, ethical approval was not required as the de-identified data was available on request from the UNICEF’s MICS website [14].

## Results

### Socio-demographic information of respondents

A total of 9,061 women responded to the question about HIV/AIDS, with a mean age of 28.8 (SD: 8.5) years. Table 1 shows the socio-demographic information of the participants. 81% of the respondents were married, 78.9% had no formal education, and 74.4% lived in rural areas.

**Table 1:** Characteristics of South Sudanese women aged 15–49 years old relating to HIV knowledge (n = 9061) and attitudes (n = 4717) towards people with HIV.

	Weighted*	95% CI
Age (years), mean (SD)	28.8 (8.5)	
** Age group, n (%)**		
*15–19*	1342 (14.8)	(14.1–15.6)
*20–24*	1589 (17.5)	(16.7–18.4)
*25–29*	2067 (22.8)	(21.9–23.8)
*30–34*	1489 (16.4)	(15.6–17.3)
*35–39*	1393 (15.4)	(14.6–16.2)
*40–44*	626 (6.9)	(6.4–7.5)
*45–49*	555 (6.1)	(5.6–6.7)
** Marital status, n (%)**		
Never married	1013 (11.2)	(10.5–11.9)
Currently married	7344 (81.0)	(80.1–81.9)
Formerly married	705 (7.8)	(7.2–8.4)
**Wealth index quintiles, n (%)**		
*Poorest*	1724 (19.0)	(18.0–20.1)
*Second*	1743 (19.2)	(18.3–20.2)
*Middle*	1796 (19.8)	(18.8–20.8)
*Fourth*	1856 (20.5)	(19.5–21.5)
*Richest*	1942 (21.4)	(20.3–22.6)
**Education, n (%)**		
*None*	7145 (78.9)	(77.8–79.9)
*Primary*	1534 (16.9)	(16.1–17.8)
*Secondary +*	353 (3.9)	(3.4–4.4)
*Adult education*	29 (0.3)	(0.2–0.5)
**Area, n (%)**		
*Rural*	6745 (74.4)	(73.2–75.6)
*Urban*	2316 (25.6)	(24.4–26.8)
**State, n (%)**		
*Upper Nile*	1088 (12.0)	(11.2–12.9)
*Warap*	1270 (14.0)	(13.1–15.0)
*Northern Bahr El Ghazal*	778 (8.6)	(8.0–9.2)
*Jonglei*	1299 (14.3)	(13.4–15.4)
*Unity*	594 (6.6)	(6.1–7.1)
*Lakes*	659 (7.3)	(6.8–7.8)
*Western Bahr El Ghazal*	323 (3.6)	(3.3–3.9)
*Eastern Equatoria*	1012 (11.2)	(10.4–12.0)
*Central Equatoria*	1261 (13.9)	(12.9–15.0)
*Western Equatoria*	777 (8.6)	(7.9–9.3)
** Ever heard of HIV/AIDS, n (%)**	9061	.
*Yes*	4816 (53.1)	(51.9–54.4)
*No*	4245 (46.9)	(45.6–48.1)
**Good knowledge, n (%)**	9061	.
*Yes*	1920 (21.2)	(20.2–22.2)
*No*	7141 (78.8)	(77.8–79.8)
**Positive attitudes, n (%)**	4717	.
*Yes*	1039 (22.0)	(20.7–23.4)
*No*	3678 (78.0)	(76.6–79.3)

* Number of valid cases is different from the total count in the crosstabulation table because the cell counts have been rounded.

### HIV/AIDS knowledge

Of all study participants, 53.1% were aware of HIV/AIDS, and 21.0% had good knowledge of HIV/AIDS. Middle-aged women (20–34 years) had relatively good knowledge of HIV/AIDS compared to younger women aged 15–19 years old (Table 2). When individual-level factors were controlled for, women aged 20–24 (AOR = 1.32; 95% CI: 1.04–1.67), 25–29 (AOR = 1.29; 95% CI: 1.02–1.64) and 30–34 (AOR = 1.30; 95% CI: 1.01–1.68) years old had significantly good knowledge than women in the 15–19 years old cohorts. However, only women aged 20–24 years old were found to have significantly good knowledge (AOR = 1.28, 95% CI: 1.01–162) after accounting for the individual/household and community level factors.

**Table 2:** Univariable and multivariable binary logistic regression for HIV knowledge (n = 9061) among women in South Sudan.

		Univariate				Model 1: Multivariable				Model 2: Multivariable			
	*n (%)*	* *	*95% CI*		* *	* *	*95% CI*		* *	* *	*95% CI*		* *
		OR	Lower	*Upper*	*p-value*	*AOR*	*Lower*	*Upper*	*p-value*	*AOR*	*Lower*	*Upper*	*p-value*
** Age**													
*15–19*	304 (22.7)	ref	ref	ref	.	ref	ref	ref	.	ref	ref	ref	.
*20–24*	402 (25.3)	1.24	1.01	1.52	0.036	**1.32**	**1.04**	**1.67**	0.020	**1.28**	**1.01**	**1.62**	0.041
*25–29*	440 (21.3)	1.11	0.91	1.35	0.295	**1.29**	**1.02**	**1.64**	0.038	1.25	0.98	1.59	0.068
*30–34*	301 (20.2)	1.06	0.86	1.31	0.586	**1.30**	**1.01**	**1.68**	0.042	1.27	0.98	1.63	0.070
*35–39*	259 (18.6)	0.90	0.72	1.12	0.332	1.17	0.90	1.52	0.231	1.13	0.87	1.46	0.376
*40–44*	111 (17.7)	0.81	0.62	1.08	0.151	1.03	0.75	1.41	0.857	0.97	0.70	1.33	0.837
*45–49*	85 (15.3)	0.66	0.49	0.89	0.007	0.89	0.64	1.24	0.494	0.83	0.59	1.16	0.265
**Marital status**													
*Never married*	245 (24.2)	ref	ref	ref	.	Ref	ref	ref	.	ref	ref	ref	.
*Currently married*	1454 (19.8)	0.99	0.82	1.19	0.889	1.25	0.98	1.59	0.070	1.23	0.97	1.57	0.092
*Formerly married*	204 (28.9)	1.16	0.89	1.51	0.267	**1.62**	**1.19**	**2.21**	0.002	**1.49**	**1.09**	**2.03**	0.012
** Wealth index quintiles**													
*Poorest*	151 (8.7)	ref	ref	ref	.	ref	ref	ref	.	ref	ref	ref	.
*Poorer*	187 (10.7)	1.09	0.85	1.40	0.501	1.06	0.83	1.37	0.631	1.03	0.80	1.33	0.825
*Middle*	293 (16.3)	**1.46**	**1.15**	**1.86**	0.002	**1.40**	**1.10**	**1.79**	0.006	1.25	0.98	1.59	0.076
*Richer*	446 (24.0)	**1.77**	**1.39**	**2.26**	0.000	**1.60**	**1.25**	**2.04**	0.000	1.27	0.99	1.63	0.059
*Richest*	826 (42.5)	**3.46**	**2.67**	**4.48**	0.000	**2.88**	**2.23**	**3.74**	0.000	**2.09**	**1.60**	**2.73**	0.000
**Education level**													
*None*	1014 (14.2)	Ref	ref	ref	.	ref	ref	ref	.	ref	ref	ref	.
*Primary*	648 (42.2)	**2.40**	**2.07**	**2.79**	0.000	**2.46**	**2.10**	**2.89**	0.000	**2.19**	**1.86**	**2.58**	0.000
*Secondary +*	229 (65.0)	**5.45**	**4.15**	**7.17**	0.000	**4.97**	**3.75**	**6.57**	0.000	**4.48**	**3.38**	**5.93**	0.000
*Adult education*	11 (38.8)	2.64	1.11	6.26	0.028	2.27	0.96	5.40	0.062	2.14	0.89	5.15	0.089
**Place of residence**													
*Rural*	1121 (16.6)	ref	ref	ref	.	.	.	.	.	ref	ref	ref	.
*Urban*	782 (33.7)	**2.83**	**2.08**	**3.83**	0.000	.	.	.	.	**1.40**	**1.12**	**1.76**	0.004
**State**													
*Upper Nile*	218 (20.1)	ref	ref	ref	.	.	.	.	.	ref	ref	ref	.
*Warap*	55 (4.3)	0.19	0.11	0.33	0.000	.	.	.	.	0.31	0.19	0.51	0.000
*Northern Bahr El Ghazal*	70 (9.0)	0.45	0.27	0.76	0.003	.	.	.	.	0.67	0.42	1.06	0.086
*Jonglei*	156 (12.0)	0.56	0.34	0.90	0.017	.	.	.	.	0.81	0.53	1.24	0.326
*Unity*	82 (13.8)	0.71	0.42	1.18	0.184	.	.	.	.	0.98	0.62	1.55	0.941
*Lakes*	94 (14.3)	0.76	0.46	1.25	0.282	.	.	.	.	1.02	0.66	1.59	0.916
*Western Bahr El Ghazal*	76 (23.6)	1.50	0.90	2.52	0.123	.	.	.	.	1.12	0.70	1.80	0.643
*Eastern Equatoria*	254 (25.1)	1.44	0.90	2.30	0.133	.	.	.	.	**1.66**	**1.10**	**2.51**	0.016
*Central Equatoria*	547 (43.4)	**3.68**	**2.34**	**5.81**	0.000	.	.	.	.	**2.50**	**1.68**	**3.71**	0.000
*Western Equatoria*	350 (45.1)	**4.24**	**2.67**	**6.75**	0.000	.	.	.	.	**3.45**	**2.30**	**5.19**	0.000

ref = reference category.

Note 1: Model 1 examines individual-level explanatory factors. Model 2 examines both individual and community level explanatory factors.

Controlling for individual-level factors for marital status, women in the ‘formerly married’ (AOR = 1.62; 95% CI: 1.19–2.21) cohorts had significantly good knowledge than women in the ‘never married’ cohorts. A similar finding (AOR = 1.49; 95% CI: 1.09–2.03) is observed for the ‘formerly married’ group. After accounting for individual-level factors, women in the middle (AOR = 1.40; 95% CI: 1.10–1.79), richer (AOR = 1.60; 95% CI: 1.25–2.04) and richest (AOR = 2.88; 95% CI; 2.23–3.74) wealth index quintiles had significantly good knowledge compared to women in the poorest wealth index quintiles. However, only women in the richest (AOR = 2.06; 95% CI: 1.60–2.73) wealth index quintiles were found to have a significantly good knowledge when both individual and community-level variables were controlled.

Women with primary (AOR = 2.46; 95% CI: 2.10–2.89) and secondary (AOR = 4.97; 95% CI: 3.75–6.57) education were found to have significantly good knowledge about HIV/AIDS than women with no education when controlled for individual-level factors. A similar finding was observed when both individual and community-level factors were accounted for–women with primary (AOR = 2.19; 95% CI: 1.86–2.58) and secondary (AOR = 4.48; 95% CI: 3.38–5.93) education.

Women in urban areas had more knowledge about HIV/AIDS than women in rural areas. They were 2.83 (95% CI: 2.08–3.83) times more likely to have good knowledge about HIV/AIDS than women in the rural areas and 1.40 (95% CI: 1.12–1.76) times when controlled for both individual and community-level variables. Controlling for both individual and community-level factors, women in Eastern Equatoria (AOR = 1.66; 95% CI: 1.10–2.51), Central Equatoria (AOR = 2.50; 95% CI: 1.68–3.71) and Western Equatoria (AOR = 3.45; 95% CI: 2.30–5.19) each had significantly good knowledge of HIV/AIDS compared to those in Upper Nile.

### Attitudes towards people with HIV/AIDS

Twenty-two percent (22.0%) of the 4,717 respondents assessed for attitudes towards people with HIV/AIDS expressed positive attitudes. When controlled for individual-level variables, women in the middle (AOR = 1.82; 95% CI: 1.23–2.69), richer (OR = 2.24; 95% CI: 1.53–3.27) and richest (OR = 3.06; 95% CI: 2.08–4.49) wealth index quintiles significantly had positive attitudes towards people with HIV/AIDS than respondents in the poorest category (Table 3). However, accounting for both individual and community-level factors found that only women in the richer (AOR = 1.60; 95% CI: 1.08–2.36) and richest (AOR = 2.02; 95% CI: 1.35–3.02) wealth index quintiles had significant positive attitudes compared to women in the poorest group.

**Table 3:** Univariable and multivariable binary logistic regression for attitudes (n = 4717) towards people with HIV among women in South Sudan.

		Univariate				Model 1: Multivariable				Model 2: Multivariable			
	*n (%)*		*95% CI*				*95% CI*				*95% CI*		
		*OR*	*Lower*	*Upper*	*p-value*	*AOR*	*Lower*	*Upper*	*p-value*	*AOR*	*Lower*	*Upper*	*p-value*
**Age**													
*15–19*	783 (16.6)	ref	ref	ref	.	ref	ref	ref	.	ref	ref	ref	.
*20–24*	903 (19.1)	1.23	0.95	1.59	0.111	1.34	0.99	1.80	0.055	1.28	0.95	1.73	0.103
*25–29*	1065 (22.6)	1.12	0.87	1.44	0.385	**1.37**	**1.01**	**1.86**	0.043	1.31	0.96	1.78	0.089
*30–34*	728 (15.4)	0.94	0.71	1.25	0.682	1.25	0.90	1.74	0.187	1.17	0.84	1.63	0.355
*35–39*	695 (14.7)	1.01	0.76	1.34	0.949	**1.40**	**1.00**	**1.96**	0.048	1.29	0.92	1.81	0.134
*40–44*	301 (6.4)	0.97	0.67	1.39	0.854	1.33	0.89	2.01	0.168	1.21	0.80	1.82	0.371
*45–49*	242 (5.1)	0.67	0.44	1.02	0.062	0.93	0.59	1.48	0.762	0.84	0.53	1.33	0.458
**Marital status**													
*Never married*	587 (12.5)	ref	ref	ref	.	ref	ref	ref	.	ref	ref	ref	.
*Currently married*	3707 (78.6)	0.84	0.67	1.06	0.140	0.95	0.70	1.27	0.711	0.94	0.70	1.27	0.687
*Formerly married*	423 (9.0)	0.97	0.70	1.34	0.848	1.13	0.78	1.65	0.518	1.05	0.72	1.53	0.813
**Wealth index quintiles**													
*Poorest*	553 (11.7)	ref	ref	ref	.	ref	ref	ref	.	ref	ref	ref	.
*Poorer*	626 (13.3)	1.43	0.94	2.18	0.094	1.36	0.89	2.08	0.152	1.17	0.76	1.80	0.477
*Middle*	825 (17.5)	**1.95**	**1.32**	**2.88**	**0.001**	**1.82**	**1.23**	**2.69**	0.003	1.41	0.95	2.11	0.092
*Richer*	1149 (24.3)	**2.60**	**1.78**	**3.79**	**0.000**	**2.24**	**1.53**	**3.27**	0.000	**1.60**	**1.08**	**2.36**	0.020
*Richest*	1565 (33.2)	**3.94**	**2.69**	**5.76**	**0.000**	**3.06**	**2.08**	**4.49**	0.000	**2.02**	**1.35**	**3.02**	0.001
**Education level**													
*None*	3073 (65.1)	ref	ref	ref	.	ref	ref	ref	.	ref	ref	ref	.
*Primary*	1294 (27.4)	**1.89**	**1.58**	**2.26**	0.000	**1.80**	**1.48**	**2.19**	0.000	**1.60**	**1.32**	**1.95**	0.000
*Secondary +*	329 (7.0)	**4.21**	**3.16**	**5.62**	0.000	**3.59**	**2.66**	**4.84**	0.000	**3.17**	**2.35**	**4.29**	0.000
*Adult education*	21 (0.4)	**5.21**	**1.87**	**14.53**	0.002	**4.24**	**1.50**	**11.93**	0.006	**3.89**	**1.37**	**11.07**	0.011
**Place of residence**													
*Rural*	3105 (65.8)	ref	ref	ref	.	.	.	.	.	ref	ref	ref	.
*Urban*	1612 (34.2)	**2.28**	**1.73**	**3.02**	0.000	.	.	.	.	**1.39**	**1.08**	**1.80**	0.011
**State**													
*Upper Nile*	518 (11.0)	ref	ref	ref	.					ref	ref	ref	.
*Warap*	265 (5.6)	0.16	0.07	0.37	0.000	.	.	.	.	0.26	0.11	0.62	0.002
*Northern Bahr El Ghazal*	320 (6.8)	0.35	0.19	0.67	0.001	.	.	.	.	0.55	0.29	1.03	0.060
*Jonglei*	497 (10.5)	0.87	0.52	1.47	0.609	.	.	.	.	1.29	0.78	2.14	0.324
*Unity*	229 (4.8)	0.34	0.16	0.70	0.004	.	.	.	.	0.48	0.23	0.97	0.040
*Lakes*	288 (6.1)	0.56	0.31	1.03	0.063	.	.	.	.	0.82	0.46	1.47	0.508
*Western Bahr El Ghazal*	197 (4.8)	1.55	0.88	2.72	0.131	.	.	.	.	1.23	0.71	2.15	0.462
*Eastern Equatoria*	535 (11.3)	**1.98**	**1.21**	**3.24**	0.006	.	.	.	.	**2.28**	**1.43**	**3.63**	0.001
*Central Equatoria*	1162 (22.6)	**2.40**	**1.53**	**3.76**	0.000	.	.	.	.	**2.04**	**1.33**	**3.12**	0.001
*Western Equatoria*	706 (15.0)	**2.17**	**1.37**	**3.46**	0.001	.	.	.	.	**2.12**	**1.36**	**3.31**	0.001

ref = reference category.

Note 2: Model 1 examines individual-level explanatory factors. Model 2 examines both individual and community level explanatory factors. Bolded text indicates significant findings.

A significant difference was also observed in attitudes toward people with HIV/AIDS between respondents with no formal education and respondents with primary and secondary education. For example, women with secondary education were 4.48 (95% CI: 3.38–5.93) times more likely to have positive attitudes towards people with HIV/AIDS than women with no education when controlled for both individual and community-level factors.

Women in urban areas were 2.28 (95% CI: 1.73–3.02) times more likely to have positive attitudes towards people with HIV/AIDS than women in rural areas. After controlling for both individual and community-level variables, women in urban areas (AOR = 1.39; 95% IC: 1.08–1.80) had significant positive attitudes towards people with HIV/AIDS compared to women in rural areas. When controlled for both individual and community-level factors, women in Eastern (AOR = 2.28; 95% CI: 1.43–3.63), Central (AOR = 2.04; 95% CI: 1.33–3.12), and Western (AOR = 2.12; 95% CI: 1.36–3.31) Equatoria states were at least two times more likely to have positive attitudes towards people with HIV/AIDS than women in Upper Nile.

### Measures of variation in HIV/AIDS knowledge and attitudes

Table 4 shows the estimates of the cluster level variations in HIV/AIDS knowledge and attitudes. Accounting for both individual/household and community level fixed effects covariates led to a significant variation in HIV/AIDS knowledge (AV = 2.11 in the null model to 0.60 in model 2) and attitudes (AV = 1.30 to 0.46 in model 2). This indicates that 71.56% and 64.62% of the contextual variation in HIV/AIDS knowledge and attitudes at the cluster-level, respectively, is attributable to the characteristics of the individual/household and community. The estimates of the intra-cluster correlation (ICC) indicate that in the fully adjusted model with both individual and community-level factors, about 15% and 12% of the total variance of HIV/AIDS knowledge and attitudes, respectively, remain unexplained. The Median Odds Ratio (MOR) indicates a 2-fold and 76% increase in the odds for HIV/AIDS knowledge and attitudes, respectively when women transitioned from below to above HIV/AIDS knowledge and attitudes in the communities.

**Table 4:** Measures of variation in HIV knowledge and attitudes.

	Null Model		Model 1		Model 2	
	*Knowledge*	*Attitudes*	*Knowledge*	*Attitudes*	*Knowledge*	*Attitudes*
**AV**	2.11	1.30	1.07	0.78	0.60	0.46
**ICC**	0.39	0.28	0.25	0.19	0.15	0.12
**MOR**	5.27	3.39	2.92	2.36	2.01	1.76
**PCV**^**#**^	-	-	49.29	40.0	71.56	64.62

AV = area variance (i.e., variance of the random effect); ICC = intra-cluster correlation; MOR = median odds ratio; PCV proportional change in variance; #null model vs model 1 or model 2.

**Fig 1:**
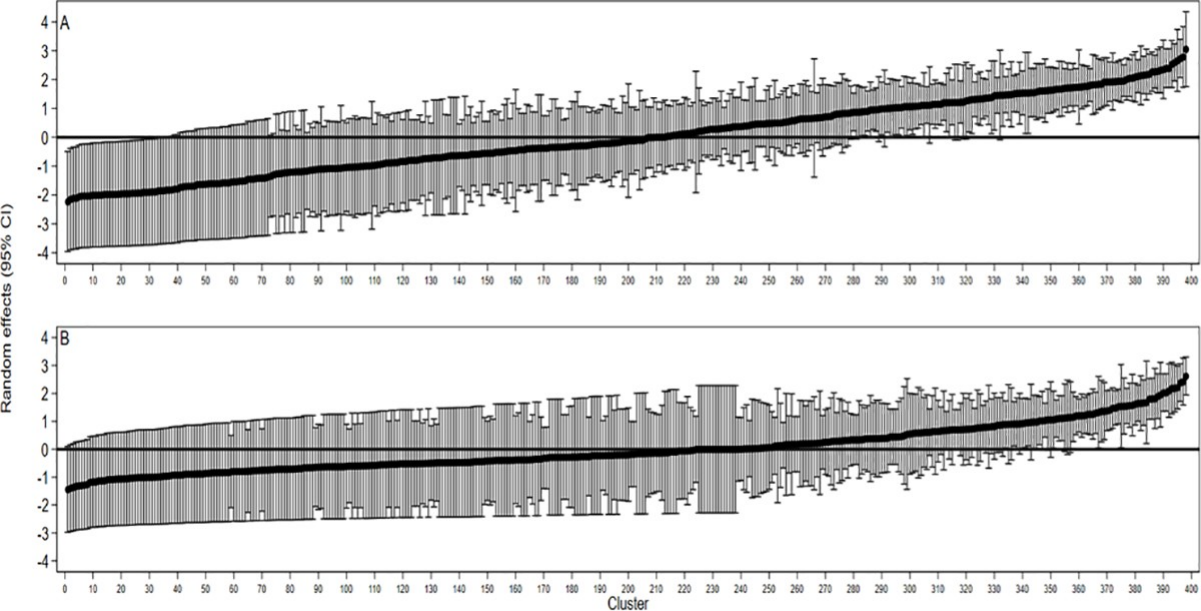
Cluster level differences in A) Knowledge level and B) Attitudes.

Fig 1 shows the caterpillar plot of the random effects with a 95% confidence interval for all 398 clusters in the sample from the null model. Clusters with confidence intervals above zero (0) signify those with an above-average level of knowledge or positive attitudes.

## Discussion

Across the world, women are known to be disproportionately affected by HIV/AIDS due to multiple vulnerability factors including socio-cultural inequity, poverty and other known social determinants of health, demanding the prioritisation of women in HIV/AIDS research [23, 24]. Women in SSA particularly remain the most disproportionately affected [25]. The current study provides insights into the knowledge and attitudes concerning HIV/AIDS among 9,061 South Sudanese women. We found that only 53.1% of the study respondents “ever heard of HIV/AIDS”. This figure is lower than in most African countries [8, 26], which could be explained by the fact that South Sudan is a fragile state and experienced protracted civil wars [1].

Social determinants, including a higher level of education and improved level of wealth, were among the factors found to increase the likelihood of women’s good knowledge and positive attitudes toward people living with HIV/AIDS. The finding shows that 71.5% of the contextual variation in HIV/AIDS knowledge at the cluster level was due to individual/household and community characteristics. This shows that factors in which people are born, work and live can shape their access to health information [23, 27]. As noted within the Sustainable Development Goals (SDG 3), addressing health literacy for women and girls is central to ensuring holistic health and addressing poverty that perpetuates HIV/AIDS in developing nations [28].

In this study, determinants such as income, education and the settings (states or rural vs urban) where respondents lived seemed to have acted together in a powerful way to shape their HIV/AIDS knowledge and attitudes [27]. An interesting observation was that people from the three states in the Equatoria region had higher odds of HIV knowledge and positive attitudes towards people living with HIV than in the other states. It is possible that the relatively high prevalence of HIV in the Equatoria region could have contributed to the differences seen in knowledge and attitudes towards people living with HIV compared to the other states [29]. The observed knowledge of HIV/AIDS is anticipated considering that South Sudan recently had independence (2011) with civil unrest and structural drawbacks [30]. As a low-income country with a fragile health system, South Sudan can enhance HIV/AIDS knowledge through collaborating with local communities to implement community-based HIV/AIDS education campaigns [31].

Twenty two percent (22.0%) of respondents expressed positive attitudes toward persons with HIV/AIDS. This finding is in line with previous evidence which reported that 90.6% of participants in South Sudan had one or more negative beliefs toward persons living with HIV/AIDS [32]. Realising that the rates of good knowledge (21.0%) are similar to positive attitudes (22.0%) suggests that interventions to yield positive attitudes toward persons with HIV/AIDS ought to address knowledge and recognise the different contextual factors that shape the uptake of public health information [31]. Marginal positive attitudes can pose critical adverse implications on treatment and spread of the virus. This is more critical for South Sudan, given the complex determinants and fragile healthcare system [30].

HIV/AIDS stigma and discrimination are well-known factors that impact individuals living with HIV/AIDS. It can prevent them from engaging actively in society, disrupting the functioning of communities and hampering HIV/AIDS prevention and treatment efforts [33]. HIV/AIDS stigma can result in some people concealing their HIV status from family members or employers due to fear of being labelled as ‘immoral’ or losing their jobs [34]. A previous cross-sectional study in South Sudan found that 70% of HIV/AIDS negative participants labelled persons living with the virus shameful and uncultured because they were thought to have multiple sexual partners [34]. Notably, women experienced higher levels of stigmatisation compared to men with more dire consequences considering patriarchal systems where women are expected, and often need, to rely on men for their basic life needs [35]. As such, negative attitudes may inhibit testing and deter persons living with HIV from disclosing their status, promoting the transmission of the virus [36]. HIV/AIDS campaigns need to highlight the stigma of persons with HIV and how such attitudes can impact prevention efforts.

Women with secondary or higher levels of education exhibited good knowledge of HIV/AIDS compared to those without formal education. Our finding supports existing literature linking good knowledge of HIV/AIDS and positive attitudes toward persons living with HIV/AIDS to high education [37, 38], indicating that education is an important determinant. Merakou, Costopoulos [39] highlighted that spending more years in education results in enhanced HIV/AIDS knowledge, which agrees with our findings. Increased education is a significant determinant of women’s empowerment and access to health information and services, which improves their capacity to navigate health systems for themselves and the people they care for [28]. Based on our findings, interventions must strive at developing HIV/AIDS knowledge while prioritising persons who have no formal education in order to bridge the knowledge and attitudinal disparity between the educated and the uneducated. This can be achieved through easily accessible mass media platforms like radio, which have proven effective in SSA [40].

Urban residents demonstrated good knowledge and positive attitudes toward persons living with HIV/AIDS compared with rural residents. This is consistent with evidence supporting the positive association between urban residence and having comprehensive HIV/AIDS knowledge and positive attitudes toward persons with HIV/AIDS [26]. Women in urban locations may have easy access to mass media campaigns and the positive net effect of social networks than rural residents [26]. For instance, in South Sudan, health infrastructure and human resource for health are skewed in favour of urban locations [41]. Urban women are more likely to have mainstream employment, education and increased exposure to health information that empowers HIV testing and management [26]. This disparity necessitates the need for concerted effort to prioritise rural locations in achieving equitable HIV knowledge and change negative attitudes toward persons with HIV/AIDS. The regional differences demonstrate the disproportionate variation in social determinants of health, including health resources and access to health information [26, 41].

### Strengths and limitations

Because of the cross-sectional design, causal effect between the socio-demographic characteristics, knowledge on HIV and attitudes toward persons with HIV/AIDS cannot be established. It is also important to acknowledge that South Sudan has been under protracted civil unrest which may also have had impacted the quality of the collected data; hence, the findings should be interpreted cautiously. We also acknowledge that the exclusion of men from the current analysis is also a limitation as the comparison between gender towards HIV knowledge and attitudes might have demonstrated additional differences between men and women. Notwithstanding, the study provides critical findings needed to support public health measures to address inequitable health information on HIV in South Sudan. The findings can be used to guide public health measures that empower women in South Sudan with HIV/AIDS knowledge. The use of a national dataset with a response and a completion rate of 96% and 78% respectively shows the representativeness and the validity of the data, which support the generalisation of our findings.

### Conclusions and recommendations

Despite significant efforts made in SSA towards the UNAIDS 90-90-90 targets by 2020, it is obvious that the low HIV/AIDS knowledge among women as demonstrated in this paper will continue to hamper the efforts and reduce achievement that could potentially be made addressing HIV in this nation. The current study findings demonstrate that women in South Sudan generally have less knowledge and negative attitudes toward persons living with HIV/AIDS. Our study shows that women’s HIV/AIDS knowledge and attitudes toward people living with HIV in South Sudan are shaped by the social determinants of health, including poverty, education, and geographical settings where they live. Additionally, given the protracted civil conflicts that have existed in South Sudan, different structural, cultural, and political factors would have come into play across different groups of women impacting access to health information. It is also reasonable to hypothesise that HIV interventions that may have been provided to South Sudan may have disproportionately been delivered for urban and educated, with the poor rural and uneducated communities been left out which is a health inequity issue. Our own experiences inform that HIV interventions and campaigns, are most often prepared and delivered from urban settings and do not improve the health outcomes of the rural populations, putting rural populations at a disadvantage relative to their urban counterparts. We also draw from the findings for evidence of the necessity of developing policies and practices that address socioecological issues including culture, education and poverty to narrow the widening knowledge gap between urban and rural residents as well as educated against non-educated women. Our findings also provide evidence for health sector policies and programmes to enhance women and girls’ empowerment to improve literacy level and specifically to develop gender-based access to health interventions, including addressing structural interventions aimed at reducing gender inequities. Additionally, frameworks such as human rights and in mobilising communities will contribute to health equity. Removing gender-based health inequities and protecting the rights of all people are crucial steps to achieving universal access goals and health-related Sustainable Development Goal targets.

Additionally, understanding the South Sudan women’s attitude and knowledge towards HIV/AIDS and its impact is vital to support public health measures to control infection. The National Strategic Plan (NSP) for HIV/AIDS Strategic framework prioritises targeted approaches to HIV and AIDS awareness and support in South Sudan. To address the policy recommendations, an HIV/AIDS Communication Strategy is urgently required to brand and position communication programming in terms of knowledge, skills and self-efficacy, capacity, coordination, policy support, and utilisation of services and improve engagement within constituencies and stakeholders in South Sudan. Guidelines, protocols for engagement with the constituencies and stakeholders are required to promote the synergised implementation of the strategy. The development and production of targeted information tools and materials responsive to the needs of different segments of the population and disseminated through effective channels will further add value to closing knowledge gaps, correcting misconceptions, building support, acceptance, and utilisation of HIV/AIDS services. As part of its preventive health strategy, the government should also leverage campaigns encouraging people to go for annual medical tests, treatment adherence, and peer-to-peer and inter-personal communication programs to promote testing and engage in treatment and care. Coordination and integration of communication messaging will increase coherence, synergy and strengthen the capacity of the implementing partners, and using themed strategic communication campaigns, address regional, religious, and cultural differences and needs in a way and language the people understand. Additionally, advocacy among decisionmakers and influential leaders as well as mainstreaming HIV messaging with other key messages can bring out the benefits of an all-inclusive strategic approach to increase communication uptake among all the population groups in South Sudan and policy level systems support.

These findings provide evidence for policymakers and health advocates to holistically understand the unique determinants of health situations for women in South Sudan. As such, well designed and coordinated approaches utilising humanitarian approaches, targeting rural and uneducated women are also required to achieve progress towards the 90-90-90 targets. The similar proportion between HIV knowledge and attitudes indicate that interventions aimed at improving HIV/AIDS knowledge are essential pre-requisites for achieving desirable change in attitudes towards persons living with HIV in South Sudan.
